# Using a miniaturized double-net trap (DN-Mini) to assess relationships between indoor–outdoor biting preferences and physiological ages of two malaria vectors, *Anopheles arabiensis* and *Anopheles funestus*

**DOI:** 10.1186/s12936-019-2913-9

**Published:** 2019-08-22

**Authors:** Alex J. Limwagu, Emmanuel W. Kaindoa, Halfan S. Ngowo, Emmanuel Hape, Marceline Finda, Gustav Mkandawile, Japhet Kihonda, Khamis Kifungo, Rukiyah M. Njalambaha, Damaris Matoke-Muhia, Fredros O. Okumu

**Affiliations:** 1 Environmental Health and Ecological Science Department, Ifakara Health Institute, P. O. Box 53, Ifakara, Tanzania; 2 School of Public Health, Faculty of Health Sciences, University of the Witwatersrand, Johannesburg, South Africa; 3 Department of Environmental Studies, Faculty of Science, Technology and Environmental Studies, Open University of Tanzania, Dar es Salaam, Tanzania; 4 Centre for Biotechnology Research and Development, Kenya Medical Research Institute, P.O Box 54840-00200, Nairobi, Kenya; 5 Institute of Biodiversity, Animal Health and Comparative Medicine, University of Glasgow, Glasgow, G12 8QQ UK

**Keywords:** DN-Mini trap, Human landing catch (HLC), Mosquito surveillance, Outdoor-biting, Parous mosquitoes, Inseminated mosquitoes, Ifakara, Residual malaria transmission

## Abstract

**Background:**

Effective malaria surveillance requires detailed assessments of mosquitoes biting indoors, where interventions such as insecticide-treated nets work best, and outdoors, where other interventions may be required. Such assessments often involve volunteers exposing their legs to attract mosquitoes [i.e., human landing catches (HLC)], a procedure with significant safety and ethical concerns. Here, an exposure-free, miniaturized, double-net trap (DN-Mini) is used to assess relationships between indoor–outdoor biting preferences of malaria vectors, *Anopheles arabiensis* and *Anopheles funestus*, and their physiological ages (approximated by parity and insemination states).

**Methods:**

The DN-Mini is made of UV-resistant netting on a wooden frame and PVC base. At 100 cm × 60 cm × 180 cm, it fits indoors and outdoors. It has a protective inner chamber where a volunteer sits and collects host-seeking mosquitoes entrapped in an outer chamber. Experiments were conducted in eight Tanzanian villages using DN-Mini to: (a) estimate nightly biting and hourly biting proportions of mosquitoes indoors and outdoors; (b) compare these proportions to previous estimates by HLC in same villages; and, (c) compare distribution of parous (proxy for potentially infectious) and inseminated mosquitoes indoors and outdoors.

**Results:**

More than twice as many *An. arabiensis* were caught outdoors as indoors (p < 0.001), while *An. funestus* catches were marginally higher indoors than outdoors (p = 0.201). *Anopheles arabiensis* caught outdoors also had higher parity and insemination proportions than those indoors (p < 0.001), while *An. funestus* indoors had higher parity and insemination than those outdoors (p = 0.04). Observations of indoor-biting and outdoor-biting proportions, hourly biting patterns and overall species diversities as measured by DN-Mini, matched previous HLC estimates.

**Conclusions:**

Malaria vectors that are behaviourally adapted to bite humans outdoors also have their older, potentially infectious sub-populations concentrated outdoors, while those adapted to bite indoors have their older sub-populations concentrated indoors. Here, potentially infectious *An. arabiensis* more likely bite outdoors than indoors, while potentially infectious *An. funestus* more likely bite indoors. These observations validate previous evidence that even outdoor-biting mosquitoes regularly enter houses when young. They also demonstrate efficacy of DN-Mini for measuring indoor–outdoor biting behaviours of mosquitoes, their hourly biting patterns and epidemiologically relevant parameters, e.g., parity and insemination status, without exposure to volunteers. The trap is easy-to-use, easy-to-manufacture and affordable (prototypes cost ~ 100 US$/unit).

## Background

Malaria-related deaths around the world decreased from 655,000 in 2010 to 455,000 in 2017, but these successes are fragile and could be lost if ongoing efforts are not sustained [1]. Further investments are needed to support the elimination agenda, and to monitor dynamics of malaria transmission across countries. Evidence suggests that vector control tools, mainly insecticide-treated nets (ITNs) and indoor residual spraying (IRS), have played a major role in the observed declines of malaria mortality and morbidity [2]. However, these approaches, which effectively target indoor-biting and indoor-resting mosquitoes are increasingly compromised by factors such shifts in mosquito behaviours (e.g., early-evening biting and outdoor biting), which overlap with human activities outdoors [3, 4] and the spread of mosquito resistance to common public health pesticides [5, 6].

To accelerate malaria elimination efforts and sustain the gains made, these important aspects of persistent malaria transmission must be measured in adequate detail, and effective interventions deployed to complement ITNs and IRS. The World Health Organization (WHO) recently outlined key indicators that should be assessed to comprehensively understand the prevailing malaria transmission and its vectors in any given area [7]. These include when and where biting exposure occurs and whether available interventions are effective. Separately, the Global Technical Strategy for Malaria Elimination (GTS 2016–2030) recommended that countries should adopt effective malaria surveillance, not just as an add-on to ongoing control activities, but rather as a core intervention in itself [8]. To achieve this, effective and scalable tools are required that can be applicable even in low-income settings. With the growing attention on residual malaria transmission, such tools should not only to measure transmission and monitor interventions, but more importantly compare proportions of exposure in different locations, particularly indoors versus outdoors.

For decades, human landing catches (HLC), where adult male volunteers expose their legs to collect blood-seeking mosquitoes, have been the standard approach for sampling host-seeking mosquitoes [9, 10], and also the most reliable for comparing outdoor-biting versus indoor-biting behaviours of major malaria vectors. However, HLC is expensive and labour intensive, and raises many ethical concerns because of risks to human volunteers who collect mosquitoes. Alternative trapping techniques that can overcome these challenges are therefore needed. For residual transmission settings, these alternative traps should preferably be applicable both inside and outside houses.

In recent years, multiple new ways of conducting HLC safely have been evaluated. Examples include the human-baited double net (HDN), which was efficacious in outdoors settings in Southeast Asia, but is considered too large and bulky to use inside houses [11]. Another candidate, Ifakara Tent trap, also allows exposure-free mosquito sampling outdoors [12, 13], but like the HDN, is too large and too bulky to use indoors. Third is the mosquito electrocuting grid trap (MET), which was recently shown to catch more mosquitoes than HLC in Tanzania, and arguably provides high quality, epidemiologically relevant metrics of malaria transmission [13–15]. The MET is particularly good for measuring human exposure to bites, but is considerably expensive, requires constant electricity supply, and is not readily scalable in its current format. Perhaps the most common trap is the Center for Disease Prevention and Control light trap (CDC-light trap) [16], which is widely used by researchers and public health agencies [10]. Unfortunately, CDC-light traps also require augmentation with host odours, and are therefore used mainly indoors beside human-occupied nets [16]. Lastly, improved understanding of mosquito olfactory systems, have resulted in several proprietary traps applicable for both malaria and non-malaria vectors, e.g., BG-Sentinel, Suna Trap and BG-malaria traps [17, 18]. These traps are usually baited with synthetic mosquito attractants, such as Ifakara lure [19] or Mbita lure (MB5) [20] and carbon dioxide gas (CO_2_) to mimic natural host odours. A reliable, cost-effective and exposure-free technique that can be used both indoors and outdoors therefore remains elusive even as countries struggle with the atypical features of residual malaria transmission.

Entomologists typically assess times of night when people are most bitten by different vector species, percentage of bites occurring outside *versus* inside houses, and infectiousness of the mosquitoes. Such entomological data, if overlaid with observed human activities can help determine important indicators such as: (a) proportions of actual malaria exposure indoors and outdoors, and, (b) proportions preventable by specific interventions. Observations of increasing outdoor-biting have raised concerns that existing indoor-interventions, particularly ITNs and IRS will not be adequate for malaria control, especially in areas where the dominant vectors mostly bite outside houses [21]. However, it has also been suggested that even mosquitoes that bite humans outdoors have previously entered houses at least once in their lifecycle [22]. Field evidence is necessary to validate this hypothesis, but also to examine the extent to which specific interventions could differentially impact on malaria transmission by individual vector species behaviourally adapted to bite indoors, outdoors or anywhere.

The primary objective of this study was therefore to assess indoor–outdoor biting preferences of two major malaria vectors, *Anopheles arabiensis* and *Anopheles funestus*, in relation to their physiological ages (approximated by parity and insemination states), in rural Tanzanian villages where ITNs are already widely used but malaria transmission persists. To achieve this, a new miniaturized double net trap (DN-Mini), was designed and used for exposure-free assessment and characterization of proportions of mosquitoes biting indoors and outdoors. The DN-Mini is an adaptation of the original bed-net trap design [23], which was recently re-visited by Tangena et al. in Lao PDR [11]. It was tested here not as a replacement for any existing tool, but simply as a representative sampling tool.

## Methods

### Study area

**Fig. 1:**
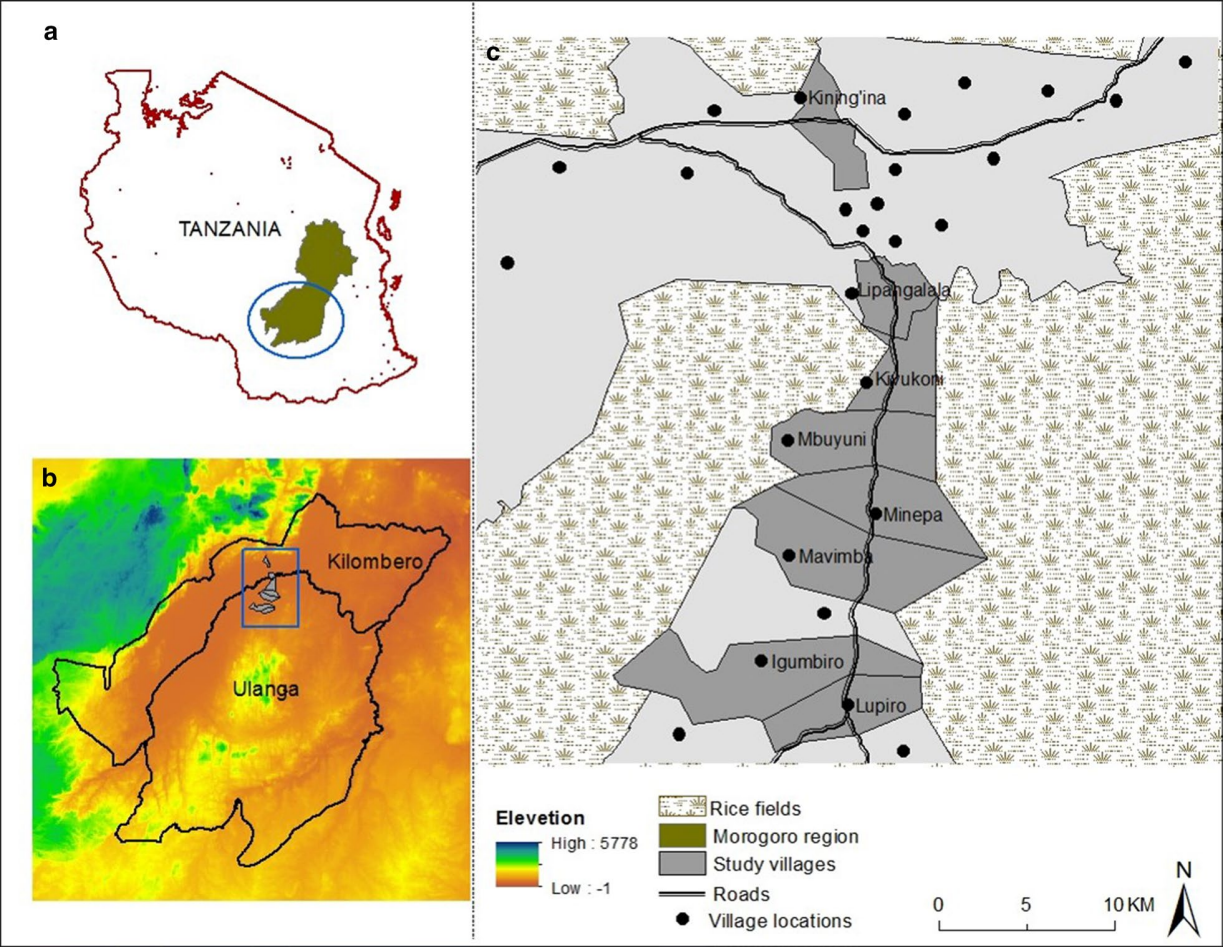
Map of the study area, showing villages in southeastern Tanzania where study was conducted

The study was done in Ulanga and Kilombero districts, south-eastern Tanzania, where ITNs are already widely used but lower-level malaria transmission persists (Fig. 1). Mosquitoes were sampled from inside and outside selected houses in eight villages of Lupiro (8.354497°S, 36.705468°E), Kining’ina (8.354497°S, 36.705468°E), Kivukoni (8.354497°S, 36.705468°E), Minepa (8.354497°S, 36.705468°E), Mavimba (8.32026°S, 36.68272°E), Mbuyuni (8.23933°S, 36.66029°E), Lipangalala (8.15304°S, 36.68481°E), and Igumbiro (8.35021°S, 36.67299°E). The area is characterized by perennial meso-endemic malaria transmission, with mosquito densities peaking between February and May [24]. The main malaria vectors comprise primarily *An. arabiensis* and *An. funestus*, the latter mediating more than 80% of transmission [25]. Long-lasting insecticide-treated nets (LLINs) are the commonest intervention against malaria and mosquito bites. Most houses in the area have mud or brick walls and either iron or grass-thatched roofs [26].

### Descriptions of miniaturized double net trap (DN-Mini)

**Fig. 2:**
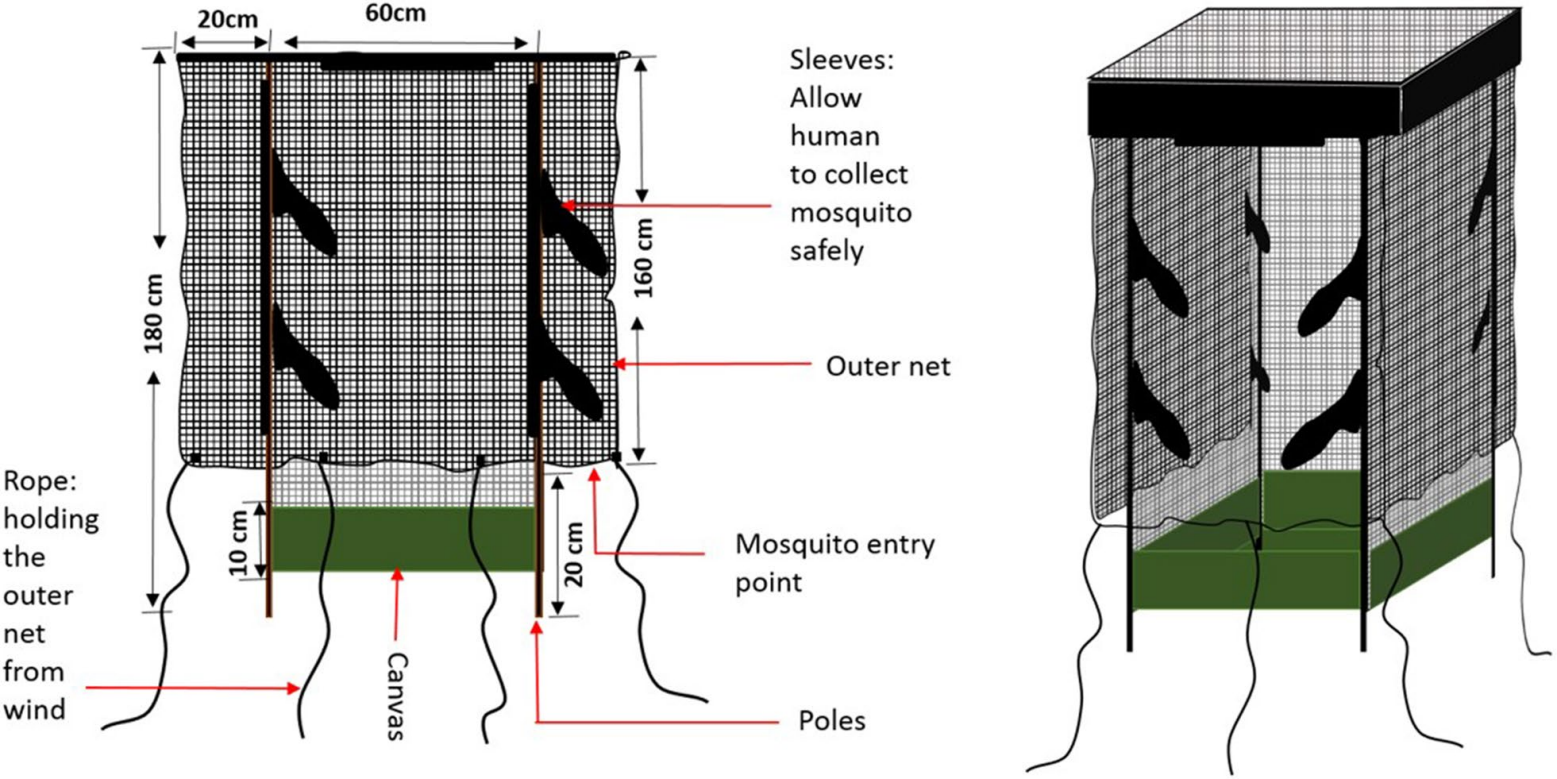
Designs and schematic drawings of the miniaturized double-net (DN-Mini), showing key features

**Fig. 3:**
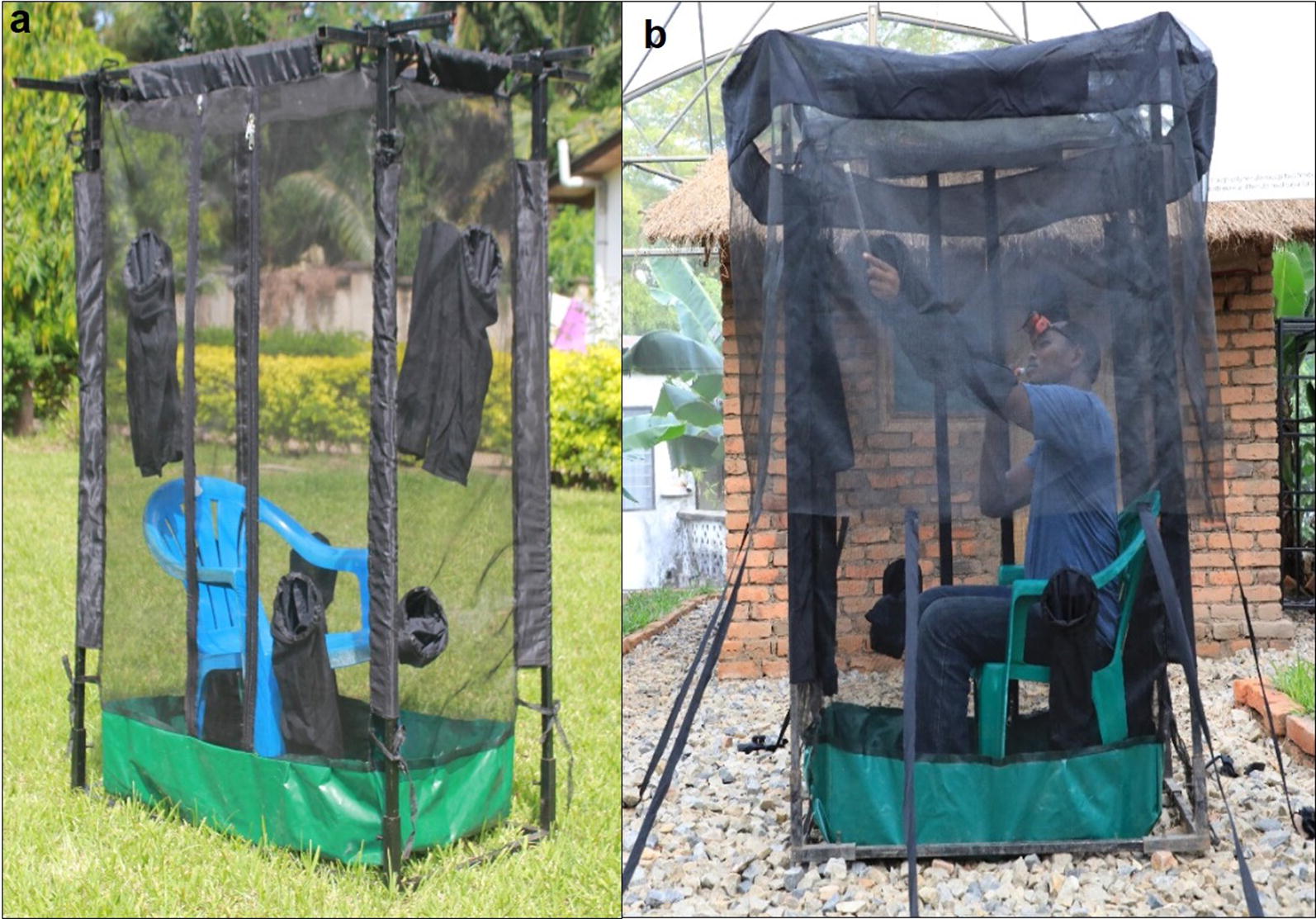
Pictorial representation of the miniaturized double-net traps (DN-Mini), showing an adult male volunteer occupying the inner compartment

The miniaturized double net trap (DN-Mini) was designed to improve comparative mosquito sampling indoors and outdoors (Fig. 2), and was not intended as a replacement for any existing tools. It is made with UV-resistant, fiber-glass netting, on a wooden (can also be metal frame) and canvas base. It has dimensions of 60 cm width, 100 cm length and 180 cm height. It has an inner protective chamber where volunteers sit to attract host-seeking mosquitoes, and a partially covered outer chamber. Host-seeking mosquitoes attempting to reach the volunteer in the inner chamber are temporarily trapped between the layers, from where they can be retrieved periodically. The inner wall has multiple sleeves through which the volunteers can safely retrieve the mosquitoes in the outer compartment using siphons (Figs. 2 and 3). Current versions of the DN-Mini cost approximately 100 US$/unit/year per unit and are made locally, but the target price is 50 US$/unit once manufacturing is scaled up. It can be assembled and dismantled by a single person in less than 5 min, and is easily moved between locations without motorized transport.

Since its development in 2016, the DN-Mini has been used to assess outdoor-biting and indoor-biting exposures to malaria vectors in and around houses fitted with spatial repellent ribbons in rural Tanzania [27]. It has also been used in assessing residual malaria transmission risk in Zanzibar (Musiba et al. unpublished) and mainland Tanzania [28]. Here, the trap is used to compare malaria vector-biting densities and nightly biting patterns between indoor and outdoor environments, which are currently best done using HLC, and to assess parity and insemination status of these mosquitoes.

### Tests to assess different DN-Mini designs with the outer layer at different heights above ground

The aim of this experiment was to determine whether varying the height above ground of the outer netting of the DN-Mini (Figs. 2 and 3), would influence the efficacy of the trap. Different versions of the DN-Mini, with the outer net at 20 cm, 50 cm or 80 cm above ground, were compared. An additional design was included of DN-Mini with the outer net at 20 cm above ground and two large holes (20 cm diameter) on the sides of the outer cover. The four DN-Mini designs were set outdoors, 150 m apart, and the traps rotated following a 4-by-4 Latin square experimental design over 12 consecutive nights, replicated in three rounds. These last tests were done in only one of the villages, Lupiro.

### Tests to compare densities of malaria vectors biting indoors and outdoors

Eight houses were identified in four of the study villages: Lupiro, Kining’ina, Kivukoni and Minepa. In each village, two houses were selected that were at least 100 m apart in each village. At each house, one DN-Mini was located indoors and another trap located outdoors, and volunteers rotated nightly between positions (Fig. 3). The traps operated from 18:00 to 07:00 h. The volunteers retrieved the mosquitoes trapped between the nets for 15 min every hour and kept them in paper cups labelled by hour. The mosquitoes were killed by freezing, then identified morphologically by taxa and sex using keys by Gillies and Coetzee [29]. Number of females of different species was recorded for each location (indoor or outdoor), house and hour of collection.

### Tests to assess species diversity, hourly biting pattern, parity status and insemination status of female mosquitoes sampled indoors and outdoors

The DN-Mini were deployed to sample mosquitoes indoors and outdoors in four of the villages, Minepa, Mbuyuni, Mavimba, and Lupiro. At each house a DN-Mini was placed indoors and another outdoors, and each of the traps occupied by adult male volunteers. The traps operated from 18:00 to 07:00, during which the volunteers retrieved mosquitoes from the DN-Mini using mouth aspirators every hour as described above. For each hourly collection, mosquitoes were packed in separate labelled cups, sorted and recorded. *Anopheles funestus* and *An. arabiensis* collected were dissected following procedures described by Detinova [30], to assess their parity status. For each hour and each location, at least 100 female mosquitoes of each species were dissected. The mosquito spermatheca were also assessed for insemination status as previously described in the WHO Manual for Practical Entomology [23], and both numbers and proportions inseminated were recorded. The parity and insemination status data were recorded for each hour of collection, both indoors and outdoors.

### Comparing DN-Mini observations of mosquito biting behaviours to observations made using human landing catches in the same villages

Since Ifakara Health Institute currently discourages the use of HLC in field studies, DN-Mini observations were compared to the last available HLC data collected indoors and outdoors in the same villages [24]. The study by Ngowo et al. [24], completed in 2016, involved trained adult male volunteers performing HLC indoors and outdoors each hour from 18:00 to 07:00 for four consecutive nights each week in the same villages. The aim of that experiment had been to assess proportions of *Anopheles* bites experienced by unprotected residents indoors *versus* outdoors. In this current study, the DN-Mini were set indoors and outdoors in four houses per village and data collected hourly to match the previous study by Ngowo et al. [24]. The indoor–outdoor biting preferences and hourly biting patterns as observed by DN-Mini were then compared to same parameters as had been observed using HLC by Ngowo et al. [24].

### Molecular identification of sibling species in the *Anopheles gambiae* complex and *Anopheles funestus* group

Since sibling species in the *An. gambiae* s.l. and *An. funestus* s.l. complexes have different biting preferences, sub-samples of mosquitoes caught indoors and outdoors in the DN Mini traps were analysed by PCR to distinguish them, using DNA extracted from hind legs. For *An. gambiae* s.l., PCR amplification was done for species-specific nucleotide sequences of ribosomal DNA (rDNA) intergenic spacer regions (IGS) in a 25-µl reaction volume of PCR mixture as described by Scott et al. [31]. For *An. funestus*, methods developed by Koekemoer et al. were used [32]. Amplification was done for the species-specific non-coding regions of the internally transcribed spacer 2 (ITS2) region on the rDNA. The post-PCR amplicons were analysed by electrophoresis in agarose gel stained with ethidium bromide. Visible DNA bands were photographed under ultraviolet light using Kodak Gel Logic 100 imaging system.

### Data analysis

Data were analysed in open sources statistical software, R version 3.5.0 [33]. Descriptive statistics were used to summarize the mosquito data and assess variance. Generalized linear mixed models (GLMM) were used to assess numbers of female mosquitoes of different species caught indoors and outdoors. Mosquito counts were modelled following negative binomial distributions to account for overdispersion in the data. The first analysis involved only the DN-Mini data, which had been collected indoors and outdoors, while the second analysis also involved HLC data which were collected both indoors and outdoors over different periods over the year. Counts of different mosquito species were included in the models as response variables, while location (indoors *vs* outdoors), the main indicator variable, was included as fixed factor. To account for variations between collection days and pseudo-replication, experimental day and household ID (nested within village) were included as random terms in the main model. Data for each mosquito species were analysed separately. Relative rates of mosquito catches, and associated 95% confidence intervals, were reported, and were considered significant when p-value was less than 0.05. All the graphs and plots were generated using a grammar for graphic package (ggplot2) [34]. The indoor–outdoor biting patterns and number of mosquitoes biting each hour were compared between the dataset obtained using DN-Mini and that originally obtained using HLC by Ngowo et al. [24].

## Results

### Densities and nightly biting patterns of mosquitoes caught indoors and outdoors by DN-Mini or human landing catch

A total of 8560 mosquitoes were collected both indoors and outdoors using the DN-Mini. Of these, 93.8% (n = 8033) were culicines (*Culex* spp. and *Mansonia* spp.), while 6.2% (n = 527) were *Anopheles*. Among the *Anopheles*, 80.6% (n = 425) were *An. arabiensis,* 16.3% (n = 86) were *An. funestus* s.l and the remaining 3.1% (n = 16) were a mixture of *Anopheles coustani, Anopheles ziemanni* and *Anopheles pharoensis*. More than twice as many *An. arabiensis* were caught outdoors as indoors (p < 0.001, Table 1). For *An. funestus,* the indoor catches were marginally higher than outdoors, though these differences were not statistically significant (p = 0.201, Table 1). Catches of the non-malaria vectors, *Mansonia spp.* were also higher outdoors than indoors, while catches of *Culex* spp. were similar between the positions (Table 1).

**Table 1:** Mean numbers of female mosquitoes of different species caught indoors and outdoors by the two different mosquito trapping methods, i.e. Miniaturized Double Net trap (DN-Mini) and Human Landing Catches (HLC) on hourly basis

Species	Location	Miniaturized double net trap (DN- Mini)			Human landing catches (HLC)		
		Mean ± SE	RR (95% CI)	p-value	Mean ± SE	RR (95% CI)	p-value
*Anopheles arabiensis*	Indoor	1.17 ± 0.70	1		12.25 ± 2.38	1	
	Outdoor	3.23 ± 1.92	2.30 [1.44–3.70]	< 0.001	25.99 ± 4.26	2.30 [1.99–2.67]	< 0.001
*Anopheles funestus*	Indoor	0.51 ± 0.28	1		1.49 ± 0.53	1	
	Outdoor	0.33 ± 0.16	0.69 [0.39–1.22]	0.201	0.99 ± 0.22	1.05 [0.81–1.38]	0.703
*Mansonia* species	Indoor	0.12 ± 0.09	1		4.30 ± 0.90	1	
	Outdoor	3.03 ± 1.09	24.43 [11.74–50.83]	< 0.001	7.48 ± 1.49	1.71 [1.42–2.07]	< 0.001
*Culex* species	Indoor	30.12 ± 7.13	1		77.51 ± 10.02	1	
	Outdoor	32.07 ± 7.02	1.07 [0.84–1.37]	0.593	66.76 ± 8.89	0.87 [0.80–0.95]	< 0.01

The relative rates (RR) as well as standard errors (SE) of the means are included

In comparison, 61,093 mosquitoes were collected indoors and outdoors using HLC in the earlier study by Ngowo et al. [24], of which 79.06% (n = 48,300) were culicines and 20.94% (n = 12,793) were *Anopheles*. Of the *Anopheles* mosquitoes collected, 92.05% (n = 11,776) were *An*. *arabiensis. An. funestus* were 5.98% (n = 765), and the remaining 1.97% (n = 252) consisted of *An. coustani* and *An. pharoensis*. Similar to DN-Mini, HLC caught more than twice the number of *An. arabiensis* outdoors compared to indoors (p < 0.001, Table 1), and there were no statistically different catches of *An. funestus* outdoors compared to indoors (p = 0.703, Table 1). For the, non-malaria vectors, *Mansonia* sp. were more abundant outdoors compared to indoors (p < 0.001, Table 1) while *Culex* were marginally higher indoors compared to outdoors (p < 0.01, Table 1).

**Fig. 4:**
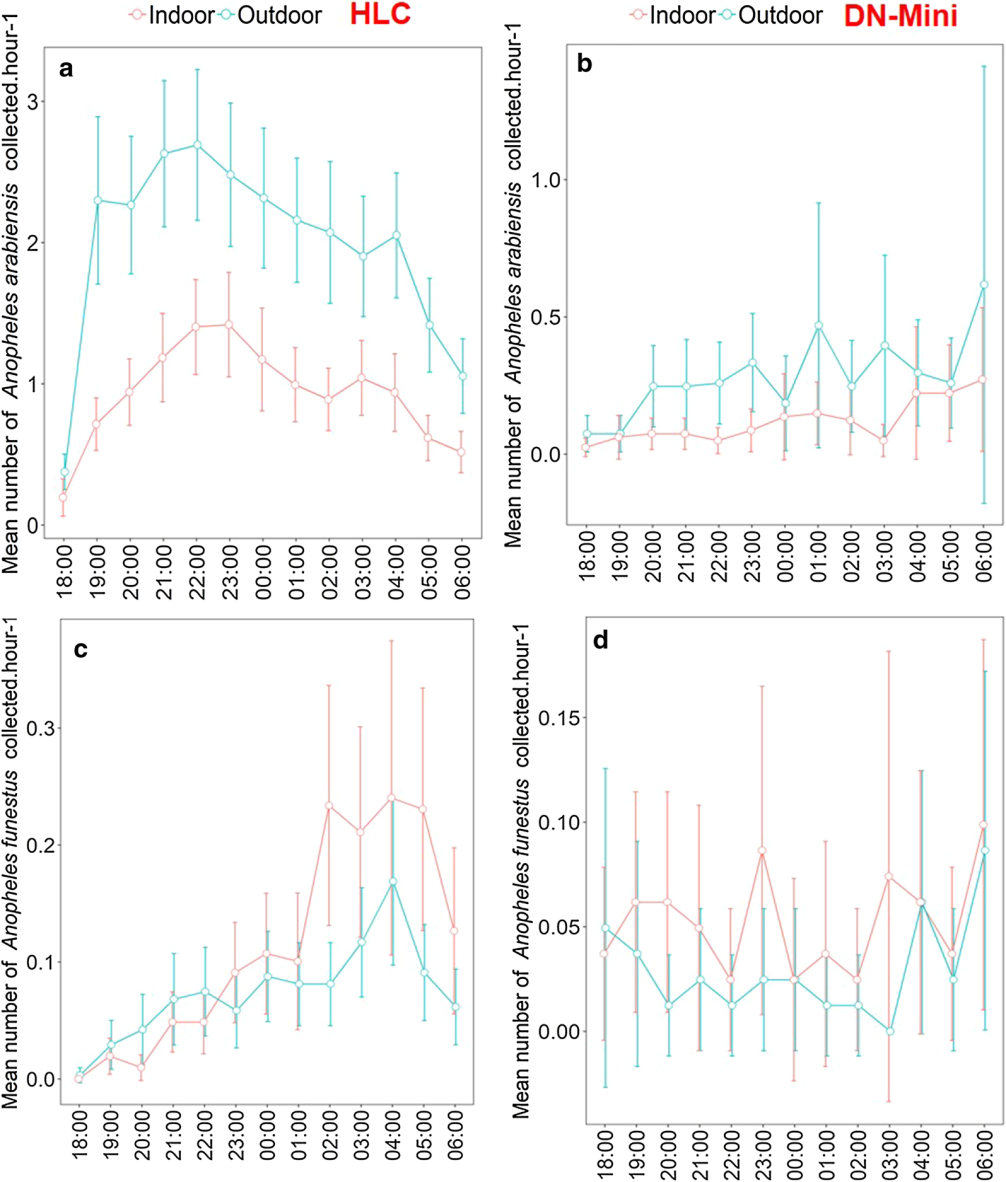
Mean number of *Anopheles arabiensis* and *Anopheles funestus* mosquitoes caught indoors and outdoors using the miniaturized double net trap (DN-Mini) or human landing catches (HLC)

**Fig. 5:**
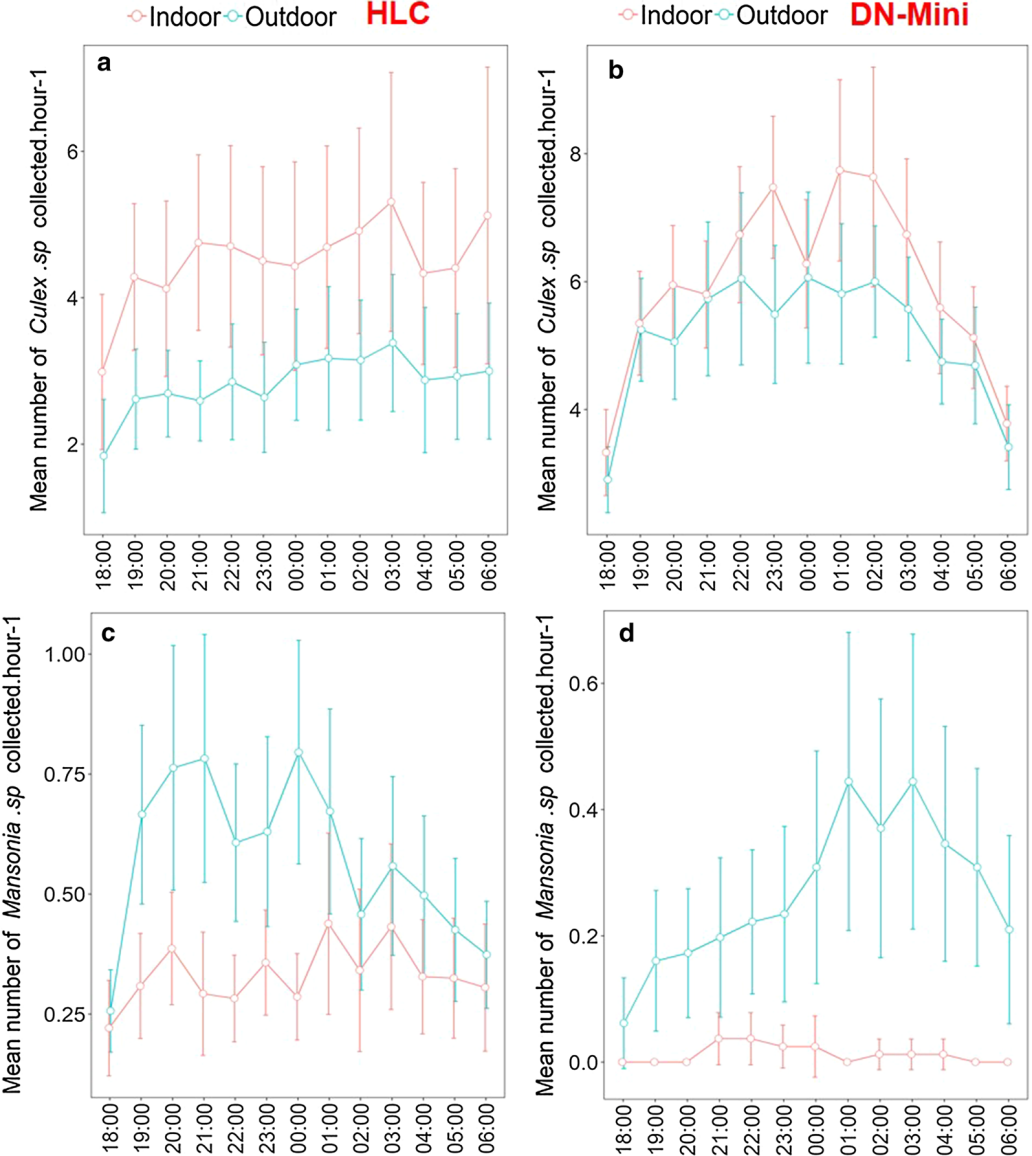
Mean number of *Culex* and *Mansonia* mosquitoes caught indoors and outdoors using the miniaturized double net trap (DN-Mini) or human landing catches (HLC)

The hourly catches were also assessed and summarized for all the nights of collection. These data showed similarity in hourly biting patterns between DN-Mini and HLC (Figs. 4 and 5), in both indoor and outdoor data. HLC data described in this section are from the previous study by Ngowo et al. [24].

### Proportions of parous mosquitoes indoors and outdoors

**Fig. 6:**
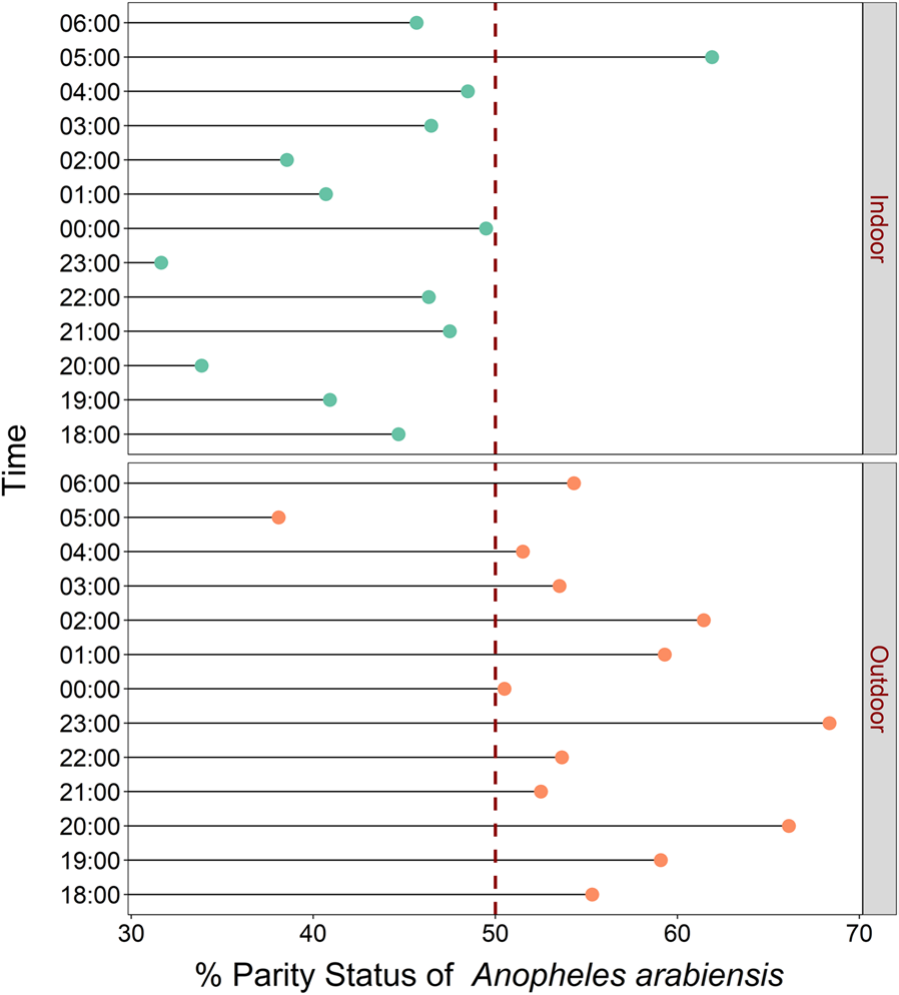
Hourly distribution of proportions of female *Anopheles arabiensis* that were parous. Data collected indoors and outdoors using DN-Mini

**Fig. 7:**
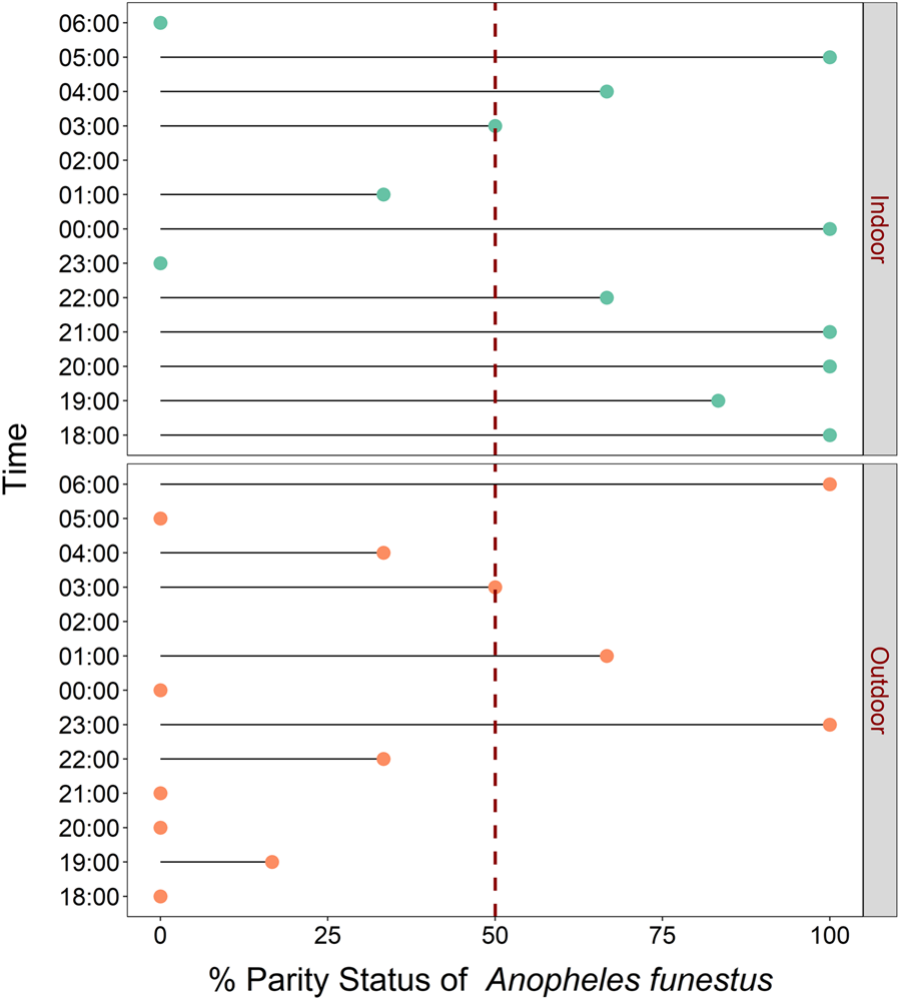
Hourly distribution of proportions of female *Anopheles funestus* that were parous. Data collected indoors and outdoors using DN-Mini

The physiological (but not chronological) age distribution of the two main malaria vector species (based on parity status as proxy) closely followed the overall biting preferences for indoors and outdoors. Data from the DN-Mini experiment showed that there was a higher proportion of parous *An. arabiensis* mosquitoes outdoors compared to indoors (t = − 3.78, df = 24, p < 0.001, Fig. 6). On the contrary, there were higher proportions of parous *An. funestus* mosquitoes indoors than outdoors, even though this difference was statistically marginal (t = 2.1335, df = 22, p = 0.04, Fig. 7). Based on parity data, *An. arabiensis* females caught outdoors were therefore generally older than those caught indoors, while for *An. funestus*, the females caught indoors were slightly older than those caught outdoors.

### Proportions of inseminated mosquitoes indoors and outdoors

**Fig. 8:**
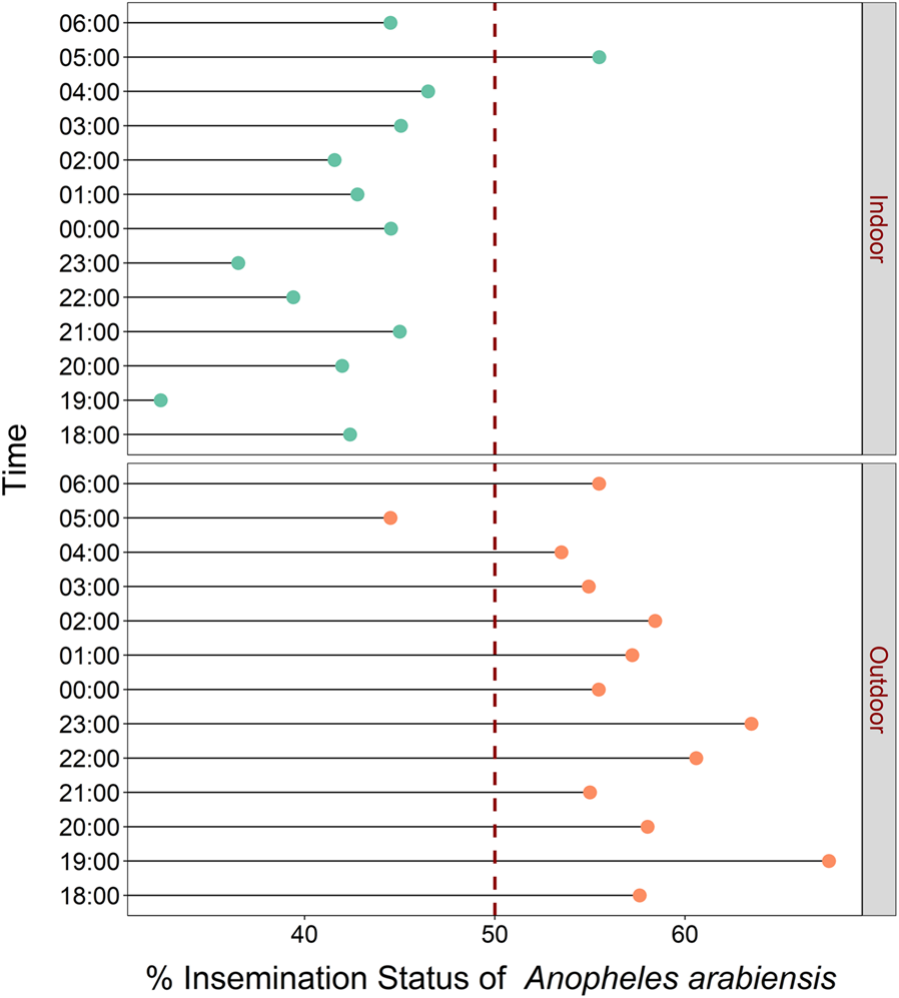
Hourly distribution of proportions of female *Anopheles arabiensis* that were inseminated. Data collected indoors and outdoors using DN-Mini

**Fig. 9:**
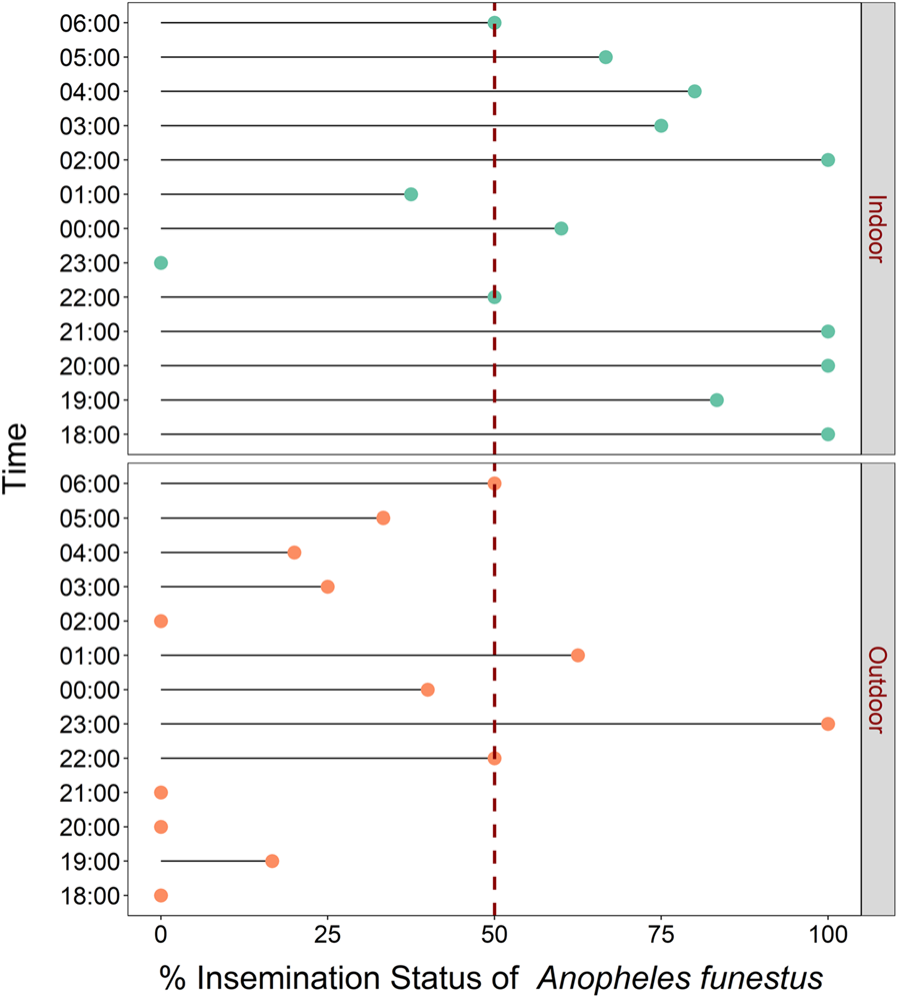
Hourly distribution of proportions of female *Anopheles funestus* that were inseminated. Data collected indoors and outdoors using DN-Mini

There were also differences in proportions of inseminated females collected indoors compared to outdoors. For both *An. arabiensis*, there was higher insemination outdoors than indoors (t = − 6.66, df = 24, p < 0.001), while for *An. funestus*, insemination was higher indoors than outdoors (t = 3.31, df = 24, p < 0.01) (Figs. 8 and 9).

### Assessing efficacy of different DN-Mini designs with the outer layer at different heights above the ground

**Fig. 10:**
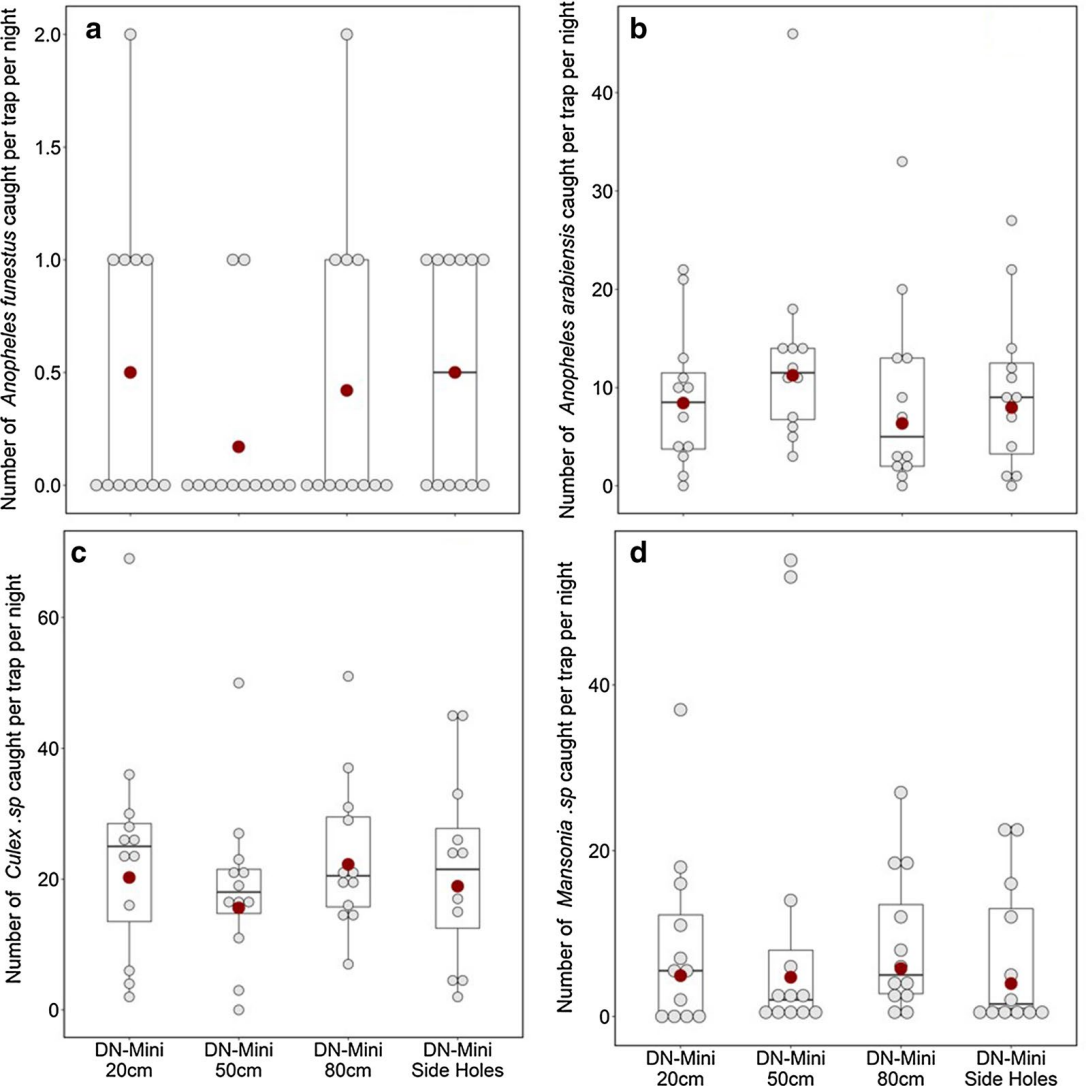
Mean nightly densities of mosquitoes caught using DN-Mini, when the outer layer was at 20 cm, 50 cm, 80 cm above ground and 20 cm but with holes

When the outer layer was 50 cm from the ground, the number of *An. arabiensis* caught was slightly higher compared to when the outer layer was 20 cm from the ground. There was however no statistical significance in the observed differences between the two settings (Fig. 10a, b). The other heights also did not yield statistically significant differences compared to the 20 cm heights (Fig. 10c, d).

### Molecular identification of *Anopheles gambiae* s.l. and *Anopheles funestus* s.l. caught indoors and outdoors by DN-Mini

The total number of *An. gambiae* s.l. examined from the outdoor and indoor collections were 478 and 574, respectively. Of all successful PCR amplifications of the *An. gambiae* s.l. mosquitoes collected either indoors or outdoors, 100% were *An. arabiensis*. Only 13 of the indoor specimens (3%) and 12 of the outdoor specimens (2%) did not amplify in the PCR tests. For *An. funestus* s.l., a total of 107 specimens from the outdoor collections and 137 from outdoor collections were examined by PCR. In the indoor collections, 57% were *An. funestus* s.s., 29% were *Anopheles rivulorum* and 14% were unamplified, while in the outdoor collections, 56.2% were *An. funestus* s.s., 30.1% were *An. rivulorum*, 0.7% were *Anopheles leesoni*, and 12.4% were unamplified.

## Discussion

Achieving malaria elimination in sub-Saharan African requires new mosquito surveillance approaches that can effectively capture important entomological parameters, including the key behaviours and physiologies of the dominant vectors. Such an approach must be safe, highly scalable, low-cost, and readily applicable across endemic settings without necessarily requiring specialized skills. In residual transmission settings where outdoor-biting can be an important contributor to transmission, the surveillance tools should particularly enable characterization of these outdoor-biting sub-populations in relation to indoor-biting sub-populations. This way, one can examine the extent to which specific interventions differentially impact malaria transmission as mediated by individual *Anopheles* species that are behaviourally adapted to bite either indoors or outdoors.

This study has provided crucial field evidence on relationships between indoor–outdoor biting preferences of two major malaria vectors, *An. arabiensis* and *An. funestus*, and their physiological ages (approximated by proportions of females found to be parous or inseminated). Specifically, in this study area, i.e., in rural south-eastern Tanzania, where ITNs are already widely used but malaria transmission persists, the study determined that potentially infectious sub-populations of *An. arabiensis* more likely bite outdoors than indoors, while potentially infectious sub-populations of *An. funestus* more likely bite indoors. These differences were statistically clearer for *An. arabiensis* for which more than twice as many biting females were caught outdoors as indoors, than *An. funestus*, for which the indoor–outdoor differences were marginal, with slightly more biting females found indoors. This is the first field illustration of such relationships between physiological age and biting preferences in these mosquito species, and may suggest an even stronger behavioural adaptation than previously thought [35–37]. The findings clearly show that malaria vectors that are behaviourally adapted to bite humans outdoors (e.g., *An. arabiensis*) also have their older and potentially infectious sub-populations concentrated outdoors. On the contrary, those vector species adapted to bite more indoors have their older sub-populations concentrated indoors. *Anopheles arabiensis* was the only member of *An. gambiae* complex caught. However, for *An. funestus*, the distribution of the two common sibling species, i.e., *An. rivulorum* and *An. funestus* s.s. was similar indoors and outdoors (i.e. 56–57% *An. funestus* s.s. and 29–30% *An. rivulorum*). The observations made are therefore applicable to both *An. funestus* s.s., which now mediates more than 80% of all malaria transmission in the study area [25], and *An. rivulorum,* which has also previously been incriminated in malaria transmission in multiple locations in East Africa [25, 38–40].

Another clear signal from these field observations is the validation of previous theoretical observations that even outdoor-biting malaria vectors regularly enter houses when young [22]. The observation of nulliparous and non-inseminated *An. arabiensis* mosquitoes host-seeking indoors, while their parous and inseminated con-specifics were outdoors suggests that these mosquitoes do indeed regularly make attempts at indoor biting especially when they are still physiologically immature adults. As previously concluded by Killeen et al. [22] and Kamau et al. [41], the findings also indicate that even these vector species could be effectively controlled by indoor interventions. In this case, for example, consistent use of new generation ITNs such as those having piperonyl butoxide (PBO) [42] and IRS could effectively control most of the mature and potentially infectious *An. funestus* as well as young immature *An. arabiensis* mosquitoes, thereby accelerating malaria elimination efforts.

Other than the primary objective, this study has also provided evidence that the newly developed exposure-free DN-Mini can effectively sample representative proportions and diversities of host-seeking mosquitoes that bite indoors and outdoors. Although the absolute catches of the different mosquito species were much lower than HLC, the actual proportions and diversities of indoor–outdoor biting females, as well as their hourly biting trends, were consistent with and representative of those most recently obtained when using HLC in the same villages. In the tests to assess biting densities indoors and outdoors, the DN-Mini caught higher number of *An. arabiensis* outdoors than indoors, and slightly higher numbers of *An. funestus* indoors than outdoors. These DN-Mini observations match the known biting preferences of these mosquito species in the study area [24], which is also influenced by widespread use of primary malaria interventions such as LLINs [43]. Although the trap cannot fully replace existing methods, such as HLC for capturing effective biting densities, it can be an effective device for capturing key behaviours such as indoor–outdoor biting and hourly biting patterns overnight, as well as associated entomological parameters such as parity, insemination and sporozoite rates in these catches.

The DN-Mini is easy-to-use, does not require external energy or electricity and can be manufactured readily with locally available materials even in rural settings. The trap also catches different mosquito species as the HLC-method, which makes it a suitable candidate for mosquito sampling in different places (Fig. 2). The DN-Mini caught significantly higher numbers of non-malaria vectors such as *Culex* species indoors than outdoors, but more *Mansonia* mosquitoes outdoors than indoors, which matches previous findings in the same area by HLC [24]. Overall, the diversity of species was generally similar between the two trapping methods, with *Culex* species being the most dominant, followed by *An. arabiensis* then *An. funestus* (Table 1).

Previously, entomologists have relied on using the HLC method as the gold standard technique for sampling host-seeking mosquitoes outdoors and indoors. Other methods have been effective in only one of the two locations. For example, CDC-light traps are effective for use indoors but not outdoors, meaning that comparative assessments of indoor–outdoor biting preferences cannot be compared directly. Others, such as Ifakara tent traps [12], are effective outdoors but are too bulky to use indoors. HLC continues to be used as an interim sampling tool while entomologists are looking for suitable and affordable sampling tools [44]. Such alternative traps do not necessarily have to catch similar mosquito numbers but should capture representative diversities and demographics of the mosquito populations. Recent developments to address these gaps include the odour-baited traps, such Ifakara Tent Traps and Suna-trap [18, 45] as well as the BG-Malaria trap and BG-Sentinel trap [19]. Others are electric traps, such as mosquito electrocuting grid trap (MET) [15], which has demonstrated substantial field efficacy comparable to or even exceeding HLC [13, 14]. One study from Lao PDR also showed that a double net trap [11], with similar designs as previously described in the 1975 WHO Entomology Manual [23], can be effective for sampling malaria mosquitoes outdoors. The trap caught higher numbers of mosquitoes than any other trapping method apart from HLC outdoors, but its size may not be suitable for comparing indoor and outdoor exposures to vector species. The DN-Mini approach described here is a miniaturization of the double net system, and allows indoor and outdoor use without exposure to human volunteers (Table 2).

**Table 2:** Mean catches of different mosquito species by different DN-Mini designs with the outer layer at different heights above the ground

Species	Mean mosquito catches per night when outer layer of DN-Mini is at different heights			
	Height	Mean ± SE	RR (95% CI)	p-value
*Anopheles arabiensis*	20 cm	8.83 ± 4.07	1	
	50 cm	13.42 ± 6.32	1.33 (0.76–2.34)	0.313
	80 cm	8.83 ± 5.52	0.75 (0.42–1.35)	0.340
	20 cm + side holes	9.75 ± 4.71	0.95 (0.53–1.68)	0.849
*Anopheles funestus*	20 cm	0.50 ± 0.38	1	
	50 cm	0.17 ± 0.22	0.33 (0.07–1.65)	0.178
	80 cm	0.42 ± 0.38	0.83 (0.26–2.71)	0.763
	20 cm + side holes	0.50 ± 0.30	1.00 (0.32–3.08)	0.999
*Mansonia* species	20 cm	8.50 ± 6.19	1	
	50 cm	11.42 ± 11.47	0.96 (0.43–2.18)	0.931
	80 cm	8.67 ± 4.83	1.18 (0.54–2.58)	0.684
	20 cm + side holes	7.08 ± 4.96	0.81 (0.36–1.78)	0.593
*Culex* species	20 cm	24.17 ± 10.10	1	
	50 cm	18.67 ± 7.14	0.77 (0.50–1.18)	0.227
	80 cm	23.42 ± 6.74	1.10 (0.72–1.68)	0.662
	20 cm + side holes	21.58 ± 8.18	0.93 (0.61–1.43)	0.753

Finally, in the experiments to assess the suitable height for the outer layer of the DN-mini, 50 cm was slightly better that the default 20 cm height, but that the other heights were not different. This may also be less crucial when volunteers can retrieve mosquitoes from the outer compartment every hour, thus minimizing chances of escaping. However, in cases where investigators wish to reduce the frequency of mosquito retrieval, it may be important to use lower heights above ground than higher heights. This can also be considered an aspect for further examination and optimization of the DN-Mini.

Overall, it is important to emphasize that the experiments presented here did not investigate whether DN-Mini can replace existing methods such as HLC for monitoring mosquito densities. This too could be explored for individual study sites by establishing statistical relationships between catches of DN-mini and the reference traps.

## Conclusion

Malaria vectors that are behaviourally adapted to bite humans outdoors also have their older, potentially infectious, sub-populations concentrated outdoors, while those adapted to bite indoors have their older sub-populations concentrated indoors. Specifically, potentially infectious *An. arabiensis* more likely bite outdoors than indoors, while potentially infectious *An. funestus* more likely bite indoors. These observations also validate previous theoretical evidence that even outdoor-biting mosquitoes regularly enter houses when young. The study also demonstrated field efficacy of DN-Mini for exposure-free mosquito sampling indoors and outdoors. The DN-Mini could be effective for measuring indoor–outdoor biting behaviours of mosquitoes, including malaria vectors, their hourly biting patterns and other associated parameters such as pathogen infection and parity status, all without any exposure to human volunteers. Overall diversity of mosquitoes was also similar in DN-Mini and HLC. The experiments did not investigate whether DN-Mini can replace existing methods such as HLC for monitoring actual mosquito densities. However, this could be explored for individual study sites after establishing statistical relationships between catches of DN-mini and the reference traps. Overall, the DN-Mini is easy-to-use, easy-to-manufacture, readily scalable, small enough for indoor and outdoor spaces, and, affordable (prototypes cost ~ 100 US$/unit/year).

## Data Availability

The dataset generated by this study is available from the corresponding author upon request.

## Abbreviations

DN: double net
UV: ultra violet
HLC: human landing catch
ITNs: insecticide-treated nets
IRS: indoor residual spraying
HDN: human-baited double net
MET: mosquito electrocuting grid trap
CDC: Center for Disease Control
PCR: polymerized chain reaction
ITS: internally transcribed spacer
GLMM: generalized linear mixed models
SE: standard errors
RR: relative rates
GTS: Global Technical Strategy
DNA: deoxyribonucleic acid
IGS: intergenic spacer regions
